# Improving retention of community-recruited participants in HIV prevention research through Saturday household visits; findings from the HPTN 071 (PopART) study in South Africa

**DOI:** 10.1186/s12874-021-01415-6

**Published:** 2021-11-08

**Authors:** N. F. Bell-Mandla, R. Sloot, G. Maarman, S. Griffith, A. Moore, S. Floyd, R. Hayes, S. Fidler, H. Ayles, P. Bock, on behalf of the HPTN 071 (PopART) study team

**Affiliations:** 1 Desmond Tutu TB Centre, Department of Pediatrics and Child Health, Faculty of Medicine and Health Sciences, Stellenbosch University, Stellenbosch, South Africa; 2 FHI 360, Durham, NC USA; 3 Department of Infectious Disease Epidemiology, Faculty of Epidemiology and Population Health, London School of Hygiene and Tropical Medicine, London, UK; 4 Department of Medicine, Imperial College London, St Mary’s Campus, London, UK; 5 Department of Clinical Research, Faculty of Infectious and Tropical Diseases, London School of Hygiene and Tropical Medicine, London, UK; 6 Zambart, Lusaka, Zambia

**Keywords:** Retention, Longitudinal studies, Population cohort, Retention strategies, Household visits, Retention methods, Participant retention, Longitudinal cohort, Community-based research, Household visit timing

## Abstract

**Background:**

Identifying successful strategies to improve participant retention in longitudinal studies remains a challenge. In this study we evaluated whether non-traditional fieldworker shifts (after hours during the week and weekends) enhanced participant retention when compared to retention during traditional weekday shifts in the HPTN 071 (PopART) population cohort (PC).

**Methods:**

HPTN 071 (PopART) PC participants were recruited and followed up in their homes on an annual basis by research fieldworkers over a 3-4 year period. The average number of successful follow-up visits, where a PC participant was found and retained in the study, was calculated for each of 3 visit schedules (early weekday shift, late weekday shift, and Saturday shift), and standardized to account for variation in fieldwork shift duration. We used one-way univariate analysis of variance (ANOVA) to describe differences in mean-successful visits and 95% confidence intervals between the shift types.

**Results:**

Data on 16 651 successful visits were included. Successful visit rates were higher when conducting Saturday visits (14.0; 95% CI: 11.3-16.6) compared to both regular (4.5; 95% CI: 3.7-5.3) and late weekday shifts (5.3; 95% CI: 4.7-5.8) overall and in all subgroup analyses (*P*<0.001). The successful visit rate was higher amongst women than men were during all shift types (3.2 vs. 1.3, *p*<0.001). Successful visit rates by shift type did not differ significantly by age, over time, by PC round or by community triplet.

**Conclusion:**

The number of people living with HIV continues to increase annually. High quality evidence from longitudinal studies remains critical for evaluating HIV prevention and treatment strategies. This study showed a significant benefit on participant retention through introduction of Saturday shifts for home visits and these data can make an important contribution to the emerging body of evidence for improving retention in longitudinal research.

**Trial registration:**

PopART was approved by the Stellenbosch University Health Research Ethics Committees (N12/11/074), London School of Hygiene and Tropical Medicine (6326) ethics committee and the Division of AIDS (DAIDS) (Protocol ID 11865). PopART was registered with ClinicalTrials.gov (registration number NCT01900977).

## Background

In 2019, 38 million people were living with HIV (PLHIV) globally, the majority of whom resided in Eastern and Southern Africa (20.7 million, 54%), and an estimated 1.7 million new HIV infections [1]. In spite of advances in HIV prevention and treatment services, antiretroviral treatment (ART) coverage in high burden settings like South Africa remains low, with 68% of PLHIV on ART, well below the WHO target of 80% [2].

There remain extensive gaps in published evidence to guide strategies for implementation of HIV treatment and prevention services in high burden settings and successful completion of studies required to provide this evidence is challenging [2]. Longitudinal studies, defined as research designs that involve repeated observations of the same individuals over a period of time [3], offer opportunities for controlled and uniform analysis of the association between measured exposures and the outcome of interest, enabling more accurate attribution of causality and are a critical component of disease prevention and treatment research [3, 4]. Longitudinal studies possess various challenges, with high rates of attrition demonstrated in published literature. To illustrate; in a cohort study by Seed et al. that recruited mother and infant pairs and evaluated childhood development, attrition rates of 62%, were recorded in a four-year follow up period [5]. In a further cohort study by Whiteman et al, which evaluated waste site exposure in Queensland Australia, high attrition rates of 53% were experienced over a 12 month follow up period [6].

Causes of attrition vary by study population and research design and the characteristics of individuals experiencing attrition are an important consideration with respect to study validity [7]. Some studies have found males and younger participants more likely to experience attrition [8–11]. Other studies have found social factors, such as lower educational and income level, to be associated with higher attrition [11]. Study designs that may not be ‘participant friendly’ and include painful clinical procedures or medication regimens that were difficult for participants to tolerate are known to negatively affect study retention [10]. Conversely, studies that include interventions that are attractive to participants, such as, access to a desirable study product or intervention, that was not routinely available, show higher retention [10].

A wide range of interventions to improve participant retention in longitudinal studies have been evaluated, with some success. A recent extensive systematic review, by Teague et al, including 141 articles from 28 different countries, reported 95 different retention strategies used in longitudinal cohort studies [12]. Retention strategies were classified as i) barrier-reduction, ii) creating a project community and iii) follow-up/reminder strategies. Barrier reduction methods were shown overall to result in a 10% increase in participant retention (*P *= 0.01) and included measures to make the study environment more participant friendly e.g. consistency of staff collection, childcare facilities and modification of data collection tools. ‘Creating a project community’ included study education and provision of appreciation gifts; however, none of these interventions was shown to have a significant impact on retention. Follow up/reminder strategies included tracing interventions (follow up phone calls, SMS reminders, tracing via alternative contacts) and again none of these was shown to have a statistically significant impact on retention [12].

Identifying successful strategies to improve participant retention in longitudinal studies remains a challenge. In this study we evaluated whether extended fieldworker shifts (after hours during the week and weekends) enhanced retention when compared traditional weekday shifts in the HPTN 071 (PopART) trial in South Africa.

## Methods

### Study setting

HPTN 071 (PopART) was a three-arm cluster randomized controlled trial implemented in 21 urban and peri-urban study communities in Zambia (12 communities) and South Africa (9 communities) between 2013 and 2018. A full description of the PopART trial design was previously published [13]. The overall goal of PopART was to assess whether an HIV prevention intervention package, including testing of all community members and linkage to HIV care and treatment, could lead to decreased HIV incidence in communities. Trial communities were randomly allocated to arms A, B or C. Arm A received the full intervention package including a comprehensive household HIV prevention package with HIV testing and linkage to care delivered by a cadre by Community HIV-care providers (CHiPs) and ART for all HIV positive individuals regardless of immune status or CD4 count. Communities in arm B received the household intervention and ART as per local guidelines. Arm C communities received standard care. From 2016 onward due to change in HIV guidelines in Zambia and South Africa arms A and B were aligned. PopART communities were defined based on the catchment areas of health care facilities providing ART. The PopART trial in South Africa was implemented in 6 communities in the Cape Metro and 3 in the Cape Winelands districts. The populations of the South African PopART communities ranged from 21,386 to 82,953 individuals, with an average of 45,780 individuals. The primary outcome HIV incidence was measured in a population cohort (PC) which included approximately 2000 participants, aged 18 to 45, from each of the 21 communities, followed up over 3 to 4 years.

### PopART PC participant selection

The PopART PC was implemented from January 2014 to July 2018. PC recruitment was undertaken at baseline (PC0) with baseline surveys completed at enrolment and participants followed-up after 12 months (PC12), 24 months (PC24) and 36 months (PC36). Additional participants were recruited at PC12 (PC12N) and PC24 (PC24N). A household census was completed prior to PopART implementation that listed and enumerated households. A random selection of households identified in the census were selected for inclusion into the PC. Thereafter, individuals residing in each selected household were enumerated. From this list of enumerated individuals in each household, an eligible individual was randomly selected for inclusion in the PC, who, if accepting study participation, signed informed consent. If the randomly selected individual did not consent to participation, another eligible household member was randomly selected for inclusion. Recruitment was aimed to enrol men and women in equal numbers.

### PopART PC follow-up and retention

Follow-up household visits were completed by research enumerators (Res) and nurses and consisted of completing a questionnaire, obtaining a blood specimen for HIV testing, and offering a HIV point of care test (POCT). The questionnaire covered socio-demographic, behavioural and HIV-related topics and was completed on an electronic data capture device (EDC), which was synced daily to a cloud-based database. Phlebotomy and POCT were completed after the interview by a trained research nurse and samples transported to laboratories for testing. The success of a household visit was defined as the successful completion of the electronic questionnaire during the household visit.

Retention of PC participants was critical for PopART. Res initially worked the traditional shift type i.e. weekdays ending at 4 pm. To increase the chance of finding participants at home, and thereby enhancing study retention, the household visit schedule was adjusted during PC12 adding additional shift types, namely a late weekday shift (ending after 4pm) and a Saturday shift (ending at 2pm).

The same research teams rotated through all shift types. With few exceptions, weekday shifts lasted eight hours, while the Saturday shift lasted five hours (see Table 1 for a list of exceptions). Once initiated, his schedule remained unchanged during the remainder of the study. The allocation of participants to early weekday, late weekday and Saturday shifts was not random. Research teams communicated with participants to determine the most convenient time for survey completion in the household. This was done prior each household visit. Therefore, the time of day for completing research activities was based on operational factors and participant availability. Research teams were systematically allocated to cover a combination of shifts which allowed research activities to be completed during early weekdays, late weekdays and Saturdays, to accommodate participant availability. During each shift type four research teams (two individuals per team) conducted follow-up household visits in each community.

**Table 1:** Duration of shift days by shift type stratified by PC round

	Early weekday shift (***n*** days)	Late weekday shift (***n*** days)	Saturday shift (***n*** days)
***PC12 (July 2015-June 2016)***			
**Duration field teams active (hours)**			
4	0	0	0
5	1	0	24
6	1	0	0
7	0	16	0
8	53	125	0
***Total days worked per shift type***	***55***	***141***	***24***
***Total hours worked per shift type***	***435***	***1,112***	***120***
***PC24 (July 2016-July 2017)***			
**Duration field teams active (hours)**			
4	1	0	0
5	1	1	17
6	0	0	0
7	0	0	0
8	30	117	0
***Total days worked per shift type***	***32***	***118***	***17***
***Total hours worked per shift type***	***249***	***941***	***85***

This table details the total number of household visits completed by the research teams stratified by PC round and shift type. *Early weekday shift* research teams ending before 4pm, *Late weekday shift* research teams ending after 4pm, *Saturday shift* research teams ending at 2pm, *PC* Population Cohort

### Study design

For this study, completed within the PopART trial, we conducted a retrospective, post-hoc, cross- sectional evaluation of the association between shift type and household visit success during PC12 and PC24 follow up visits, conducted between June 2015 and July 2017, in the 9 South African communities. Participants who were followed up at PC12 (round 2) and PC24 (round 3) in South Africa were screened for inclusion. The sample was restricted to South Africa as the operational data relating to time of household visit was not accessible for Zambia. The unit of analysis was a household visit. All household visits conducted for PC12 and PC24 were included in the analysis.

### Data sources

We used data extracted from the PopART PC survey data and from an electronic contact log. Survey data used for this study included data on all household visits conducted, detailing sex, age, PC round, community of visit, time and date of visit and whether or not the visit was successfully completed. During the implementation of the PopART PC, completion of household visits were monitored using an electronic contact log (ECL) to track the status of each household visit. ECL data included documentation of the number of household visit attempts prior to completion of a successful visit. Successful visits were excluded (dropped) from analysis if the sex of the research participant was not recorded, if the date of the household visit was missing, or if the visit was completed outside the three household visit shift types.

### Data analysis

Only data from South Africa were included in the analyses. The study protocol used in both countries was the same, however there were differences in the management of operational activities. This led to each country adopting a slightly different approach to reaching research participants.

We compared the rate of successful household visits by different shift type (Table 1). This rate was calculated as the number of successful visits per day, divided by the number of hours that the research fieldworkers were active during that particular day. Every day during the study period research teams worked according to the same shift type similar in each community, thus by standardizing by hour per day, the outcome was also standardized by community and research team. We used one-way univariate analysis of variance (ANOVA) to describe differences in mean-successful visits and 95% confidence intervals between the shift types within each of the following subgroups: males, females, age 18-24 years, age 25-34 years, age >34 years, PC12, PC24, and separately for each community triplet. Successful visits were standardized by hour for each day during the study period. To investigate if patterns changed during the study period we also report outcomes per half year (from July 2015-July 2017).

### Trial registration

PopART was approved by the Stellenbosch University Health Research Ethics Committees (N12/11/074), London School of Hygiene and Tropical Medicine (6326) ethics committee and the Division of AIDS (DAIDS) (Protocol ID 11865). PopART was registered with ClinicalTrials.gov (registration number NCT01900977).

## Results

Data on 9765 (PC12) and 6885 (PC24) participants upon completion of a successful household visit at successive PC rounds were included in this study. The majority of participants were women; 70,6% at PC12 and 71,8% atPC24. Age breakdown of participants successfully visited was similar for both PC rounds; for 18-24 years: 33,1% (PC12) and 32,4% (PC24), 25-30 years: 39,8% (PC12) and 39,7% (PC24) and > 34 years: 27,1% (PC12) and 27,9% (PC24). Of the 387 days that research fieldworkers completed follow-up visits, 220 (56.8%) and 167 (43.2%) were completed during PC12 and PC24 respectively (Table 1) during which time 16651 successful participants visits were completed (Table 2). In PC12, 55 (25.0%) days were traditional weekday shifts, 141 (64.1%) days were late weekday shifts and 24 (10.9%) days were Saturday shifts. In PC24, the majority of days was also a late weekday shift (118, 70.7%). There were a few exceptions to the standard duration of shift types (weekday shifts eight hours, Saturday shifts five hours), but most days were of standard duration: 53/55 (96.4%) of 8 hour early weekday and 125/141 (88.7%) of 8 hour late weekday shifts, and 100% of 5 hour Saturday shifts (Table 1). Overall, during PC12, the largest number of visit hours were completed during late weekday shift 1,112 (66.7%) compared to 435 (26.1%) during early weekday and 120 (7.2%) during Saturday shifts. Similarly, during PC24 the largest number of visit hours were completed during late weekday shift 941 (73.8%), compared to 249 (19.5%) during early weekday and 85 (6.7%) during Saturday shifts (Table 1).

**Table 2:** Successful follow-up visits standardized by duration and date of shift type: a comparison within subgroups

Subgroups	Early weekday shift	Late weekday shift	Saturday shift	***P*** value^*^
**Days (n)**	87	259	41	
**Duration 36 field teams active (hours)**	684	2,053	205	
**Total**				
Successful visits *n*	3,069	10,713	2,869	
Mean successful visits per hour (95%CI)	4.5 (3.7-5.3)	5.3 (4.7-5.8)	14.0 (11.3-16.6)	<0.001
**Sex: male**				
Successful visits *n*	894	2,978	942	
Mean successful visits per hour (95%CI)	1.3 (1.1-1.5)	1.5 (1.3-1.6)	4.6 (3.7-5.5)	<0.001
**Sex: female**				
Successful visits *n*	2,175	7,735	1,927	
Mean successful visits per hour (95%CI)	3.2 (2.6-3.7)	3.8 (3.4-4.2)	9.4 (7.6-11.2)	<0.001
**Age: 18-24 years**				
Successful visits *n*	1,083	3,463	916	
Mean successful visits per hour (95%CI)	1.6 (1.3-1.9)	1.7 (1.5-1.9)	4.5 (3.6-5.3)	<0.001
**Age: 25-34 years**				
Successful visits *n*	1,184	4,274	1,154	
Mean successful visits per hour (95%CI)	1.7 (1.4-2.0)	2.1 (1.9-2.3)	5.6 (4.5-6.7)	<0.001
**Age: >34 years**^a^				
Successful visits *n*	802	2,975	799	
Mean successful visits per hour (95%CI)	1.2 (0.9-1.4)	1.5 (1.3-1.6)	3.9 (3.1-4.7)	<0.001
**PC round: PC12**				
Successful visits *n*	1,961	6,137	1,668	<0.001
Mean successful visits per hour (95%CI)	2.9 (2.1-3.6)	3.0 (2.5-3.6)	8.1 (5.3-10.9)	
**PC round: PC24**				
Successful visits *n*	1,108	4576	1,201	
Mean successful visits per hour (95%CI)	1.6 (0.9-2.3)	2.2 (1.8-2.7)	5.9 (2.9-8.8)	<0.001
**Study period: July – December 2015**				
Successful visits *n*	1,630	4,505	1,110	
Mean successful visits per hour (95%CI)	2.4 (1.6-3.1)	2.2 (1.7-2.8)	5.4 (2.4-8.4)	<0.001
**Study period: January – June 2016**				
Successful visits *n*	331	1,632	558	
Mean successful visits per hour (95%CI)	0.5 (0.2-0.7)	0.8 (0.6-1.0)	2.7 (1.3-4.1)	<0.001
**Study period: July – December 2016**				
Successful visits *n*	868	1,817	537	
Mean successful visits per hour (95%CI)	1.3 (0.6-1.9)	0.9 (0.5-1.2)	2.6 (0.3-4.9)	<0.001
**Study period: January – July 2017**				
Successful visits *n*	240	2,759	664	
Mean successful visits per hour (95%CI)	0.4 (0.1-0.6)	1.3 (1.0-1.7)	3.2 (0.9-5.5)	<0.001
**Community: Triplet 1**				
Successful visits *n*	897	3,631	906	
Mean successful visits per hour (95%CI)	1.3 (1.0-1.6)	1.8 (1.6-1.9)	4.4 (3.5-5.4)	<0.001
**Community: Triplet 2**				
Successful visits *n*	1,143	3,944	1,138	
Mean successful visits per hour (95%CI)	1.7 (1.3-1.9)	1.9 (1.7-2.2)	5.6 (4.5-6.6)	<0.001
**Community: Triplet 3**				
Successful visits *n*	1,069	3,138	825	
Mean successful visits per hour (95%CI)	1.5 (1.2-1.8)	1.5 (1.3-1.7)	4.0 (3.1-4.9)	<0.001

*ANOVA test was done to calculate if there was a statistically significant difference between the mean successful visits of each shift type within a subgroup. Successful visits were standardized by hour for each day during the study period

^a^The eldest participant was 46 years, therefore the number of subgroups was restricted to three age groups. One individual had age missing

Data on 391/ 17042 (2.3%) of successful visits were excluded from analysis, as a result of missing data on sex (125 visits missing gender classifications, most likely as a result of data entry error or unsuccessful synching and if the visit was completed outside of the visit shift schedule i.e. 266 visits). Overall data on 16,651 successful follow up visits completed during PC12 and PC24 was included in this analysis. Of visits included in the analysis: 3,069 (18.4%) were completed during a traditional early weekday shift, 10,713 (64.3%) during a late weekday shift and 2,869 (17.3%) during a Saturday shift (Table 2). The mean number of successful visits per hour was 4.5 (95%CI: 3.7-5.3) for traditional early weekday, 5.3 (95%CI: 4.7-5.8) for late weekday and 14.0 (95%CI: 11.3-16.6) for Saturday shifts (*P*<0.001) (Table 2).

Subgroup analyses comparing successful visits rates for different shift types was completed. When stratified by PC round, results were similar, with successful visit rates lowest during standard weekday shifts and highest on Saturdays, for both PC12 (*P*<0.001) and PC24 (*P*<0.001), the same trend was observed between both males (*P*<0.001) and females (*P*<0.001) with the successful visit rate lowest during weekday shifts and highest during Saturday shifts. Notably, the successful visit rate was higher for females compared to males for all shift types, early weekday shift (3.2 vs. 1.3, *p*<0.001), late weekday (3.8 vs. 1.5, *p*<0.001) and Saturday (9.4 vs. 4.6, *p*<0.001) shifts. When stratifying by age, successful visit rates were similar for early weekday and late weekday shift but consistent higher during Saturday shift (*P*<0.001) for all age groups.

When looking at 6 month periods from July 2015 through to July 2017 the successful visit rates were similar when comparing weekday and late weekday shifts and consistently higher on Saturday visits there was no clear trend in Saturday successful visit rates over time. Stratification by community triplet yielded similar results with similar successful visit rates when comparing weekday to late weekday shifts and consistently higher successful visit rates on Saturdays with no clear trend over time.

## Discussion

Participant retention was a key challenge in the PopART trial with an overall retention rate of 72% on the final annual round [14]. This study showed a positive impact on retention with higher successful visit rates when conducting Saturday visits compared to both regular and late weekday shifts overall and in all sub group analyses. Successful visits rates were higher during late household visits compared to regular weekday shifts, but this difference was not statistically significant. The successful visit rate was > 2 times higher amongst women than men during all shift types. This finding combined with greater challenges recruiting men outline the difficulties in recruiting and retaining men [12], and can be a specific consideration for researchers, particularly where a balance between males and females is important for study outcomes. Successful visit rates by shift type did not differ significantly by age, over time, by PC round or by community triplet. A wide range of strategies have been reported to improve retention in longitudinal studies [12, 15]; however, we did not find any previous publications evaluating the impact of adjusted working hours. The finding that additional household visits are required to successfully access men compared to women fits with previous published data on study retention [16–19]. The results of this study, which showed no significant difference in successful visit rates across age groups, is in contrast to some previous studies that reported increased difficulties accessing younger individuals for study retention [11, 16, 20, 21].

Further to adjusted working hours, we used other standardised approaches to enhancing retention including extensive use of cell phone messaging and carefully screening out individuals who reported that they were likely to move location within the study period. In published reports, targeted incentives have been identified as the most commonly documented approach to improving retention outcomes. In a systematic review by Brueton et al. [15], of 38 RCTs, monetary incentives were successful for increasing the rate of completion and or return of both postal and electronic questionnaires. Offer of a cash voucher was shown to increase return of postal questionnaires and biomedical test kits [15]. A further systematic review by Booker et al also found incentives to be an effective mechanism for improving retention with bigger incentives having a bigger positive impact [22]. We did not compensate participants for study visits and made limited use of providing incentives such as gifts (mugs, calendars etc.) and this is a strategy that can be carefully explored in future research projects.

### Study strengths and limitations

The PopART trial, one of the largest CRTs, ever completed provided a unique opportunity to evaluate participant retention in > 18 000 participants enrolled in South Africa. PopART communities differed extensively socioeconomically and with respect to urban vs. rural environments and this together with the non-random allocation of participants to different types of households’ visits *and the post-hoc nature of this analysis* is a significant limitation. The results of this study however were consistent across groups (sex, age, PC round) in stratified analysis strengthening the external validity of study results and the study was completed within a highly regulated CRT (PopART), with extensive prospective definition of interventions and procedures, as well as data quality control, which also contributed to study validity. Overall, given the size and strengths of the PopART trial we believe the results of this study can be of significant value to researcher conducting comparable longitudinal studies in high burden settings, across communities, which supported the findings being generalizable to other comparable settings.

## Conclusions

The number of people living with HIV continues to increase annually. High quality evidence from longitudinal studies remains critical for evaluating HIV prevention and treatment strategies. This study showed overall lower retention amongst male participants as well as a significant benefit on participant retention through introduction of Saturday shifts for home visits on both men and women of all age groups. This data can make an important contribution to the emerging body of evidence for improving retention in longitudinal research.

## Data Availability

The data that support the findings of this study are available from Scharp but restrictions apply to the availability of these data, which were used under license for the HPTN 071 study, and so are not publicly available. Data are however available from Scharp upon reasonable request through deborah@scharp.org. Research data has been stored and managed as per Ethics requirements

## Abbreviations

HIV: Human immunodeficiency virus
PLWHIV: People Living with HIV
PopART: Population Effects of Antiretroviral Therapy to Reduce HIV Transmission
EDC: Electronic Data Capture Device
PC: Population Cohort
PC12: Population Cohort month 12
PC24: Population Cohort month 24
ECL: Electronic Contact Log
CRT: Community-randomized trial
POCT: Point of Care Test
